# Mapping evidence on the risk factors associated with pediatric cancers in sub-Saharan Africa: a scoping review

**DOI:** 10.1186/s13643-022-01931-6

**Published:** 2022-04-04

**Authors:** Sehlisiwe Ndlovu, Mbuzeleni Hlongwa, Themba Ginindza

**Affiliations:** 1 Discipline of Public Health Medicine, School of Nursing and Public Health, University of KwaZulu-Natal, Durban, South Africa; 2 Burden of Disease Research Unit, South African Medical Research Council, Cape Town, South Africa

## Abstract

**Background:**

The rarity and heterogeneity of pediatric cancers make it difficult to assess risk factors associated with the development of cancer in this group. This also determines the quantity and quality of evidence for etiological factors linked to pediatric cancers. Evidence on the risk factors associated with pediatric cancers is scarce; however, it has been accumulating slowly over the years. As the disease burden shifts from communicable to non-communicable diseases, most of these low- to middle-income countries (LMICs) find themselves overburdened with changing health care priorities and needs. In sub-Saharan Africa, it is of major importance to pay particular attention to risk factors associated with pediatric cancer.

**Objective:**

To map evidence on risk factors associated with pediatric cancers in sub-Saharan Africa (SSA).

**Methods:**

This review was guided by Arksey and O’Malley’s framework for conducting scoping reviews. Four electronic databases were searched in December 2018, and another manual search was conducted in February 2022 to include newly published eligible articles. The databases searched included PubMed and Health Source: Nursing/Academic Edition. We also searched articles from an academic search engine, Google scholar. This review included articles reporting the relevant outcomes of this study and articles reporting cancers in children in the 0–15 years age range. This review followed the Preferred Reporting Items for Systematic Reviews and Meta-Analyses (PRISMA) extension for scoping reviews (PRISMA-ScR): checklist and explanation.

**Results:**

We retrieved 7391 articles from the initial database. The final number of studies that were included for data extraction was 15. Evidence from the retrieved studies suggests that most childhood cancers in the SSA region are infection-induced. The type of cancer mostly reported is Burkitt Lymphoma and is diagnosed mostly in the tropical region of SSA. The type of risk factors was divided into three types: infection-induced, genetic, and demographic risk factors. Overall, based on the articles retrieved, there was limited evidence on the risk factors associated with pediatric cancers in SSA.

**Conclusion:**

The limited evidence on the risk factors coupled with the lack of evidence on the true burden of these malignancies in the SSA hampers efforts to set priorities for childhood cancer control. Formulation of effective preventative (where possible) measures and treatment regimens will need proper assessment of risk factors.

## Background

Cancers are presumed to be “multivariate, multifactorial diseases that occur when a composite and prolonged process involving genetic and environmental factors interact in a multistage sequence” [1]. Disparities in childhood cancer incidence are as a result of differences to exposure to certain risk factors [2]. The rarity and heterogeneity of pediatric cancers make it difficult to assess risk factors associated with the development of cancer in this group [3–5]. This also determines the quantity and quality of evidence for etiological factors linked to pediatric cancers. Evidence on the risk factors associated with pediatric cancers is scarce [2]. Evidence with reference to causal associations of childhood cancer has been considered to be accumulating slowly in the context of pediatric cancer epidemiology [3]. The weight of evidence on the risk factors can be known, suggestive, or limited [6].

The rarity of childhood cancers has led to the poor understanding of childhood cancer etiology [7]. Accumulation of case control studies and genomic advancement technology has led to a better understanding of the factors associated with childhood cancers. Epidemiologists have suggested that prenatal and perinatal exposures may be contributing factors in pediatric cancer pathogenesis as most children present with cancer in the first years of life [7]. Factors that have been consistently associated with pediatric cancers include birth weight, parental, birth defects, and common genetic variation [3].

Environmental risk factors have been linked to pediatric cancers; however, there are only few and it is difficult to distinguish those that are causally associated from those due to bias using observational epidemiology [3]. Moreover, infectious agents like human immunodeficiency virus (HIV), Epstein-Barr virus (EBV), and human herpesvirus 6 (HHV-6) have been shown to play a crucial role in cancer development [8].

In most low- to medium-income countries (LMICs), priorities for health have paid attention to the treatment and prevention of communicable diseases, maternal, and infant mortality due to the high disease burden of such conditions and cost-effective interventions [9, 10]. As the disease burden shifts from communicable to non-communicable diseases, most of these LMICs find themselves over burdened with changing health care priorities and needs [9]. In sub-Saharan Africa (SSA), it is of major importance to pay particular attention to risk factors associated with pediatric cancer, given the high levels of cancer-related morbidity and mortality. This will not only aid in disease prediction and prevention, but also cut down on health-related costs as a result of poor health outcomes. This scoping review aimed to assess the evidence on risk factors associated with pediatric cancers in SSA.

## Methods

### Design

A scoping review study was chosen as the appropriate method to map the evidence on the risk factors associated with pediatric cancers in SSA. The Arksey and O’Malley framework for conducting scoping reviews was used to guide the review, and the steps were listed a priori in the published research protocol [11]. The research protocol outlined the reasoning or rationale and planned methods for mapping the evidence on the distribution of pediatric cancer in SSA, and the current review is based on the stated sub-research question that aimed to map evidence on the risk factors associated with pediatric cancers. The study used an amended PEO (Population, Exposure, and Outcome) framework to determine the eligibility of the research question. Only published literature was included in the review, and we aimed to include studies conducted using quantitative methods as we focused on numerical/statically derived evidence from the studies. Since this study utilized a secondary synthesis of data, which is already in the public domain, ethical approvals and consent to participate were not necessary.

### Search strategy

The literature search was conceptualized and developed under the supervision and guidance of an epidemiologist and a senior lecturer with the School of Nursing and Public Health, University of KwaZulu-Natal, South Africa. We searched articles published in PubMed, Academic search complete and Health Source: Nursing/Academic Edition. We also conducted a manual search on an academic search engine, Google Scholar. The search was carried out in December 2018 (Table 1), and another search was conducted in February 2022 to include newly published articles. We used the following keywords/search terms (“children,” “pediatric,” “childhood,” “cancer,” “pediatric cancers,” “childhood cancers,” “risk factors,” and sub-Saharan African country names and truncated terms such as “east-Africa” or “west-Africa” were also used to ensure that articles indexed using SSA country-specific names or regional terms were retrieved. The keywords were modified or changed according to the database setup. Medical Subject Headings (MeSh) were included in the search. To separate keywords during the database search, Boolean terms (AND, OR) were used. There was no language or date restrictions in the search. We anticipated to retrieve articles written in other languages besides English and had planned to utilize the services of a translator. However, all articles retrieved were written in English. Reference sections of eligible studies were reviewed to identify additional relevant articles.

**Table 1:** Database search terms

Database	Keywords	Number of studies
Pubmed	((((((“Childhood”[Journal] OR “childhood”[All Fields]) AND (“neoplasms”[MeSH Terms] OR “neoplasms”[All Fields] OR “cancer”[All Fields])) OR ((“pediatrics”[MeSH Terms] OR “pediatrics”[All Fields] OR “paediatric”[All Fields]) AND (“neoplasms”[MeSH Terms] OR “neoplasms”[All Fields] OR “cancer”[All Fields]))) AND (“risk factors”[MeSH Terms] OR (“risk”[All Fields] AND “factors”[All Fields]) OR “risk factors”[All Fields])) AND (“epidemiology”[Subheading] OR “epidemiology”[All Fields] OR “prevalence”[All Fields] OR “prevalence”[MeSH Terms])) OR (“mortality”[Subheading] OR “mortality”[All Fields] OR “mortality”[MeSH Terms])) AND (“epidemiology”[Subheading] OR “epidemiology”[All Fields] OR “morbidity”[All Fields] OR “morbidity”[MeSH Terms])) AND trend[All Fields] AND (“africa”[MeSH Terms] OR “africa”[All Fields])	7311
Health Source: Nursing/Academic Edition, Medline Academic search complete	(childhood AND cancer OR neoplasms AND pediatrics OR paediatric AND cancer OR neoplasm AND risk factors AND epidemiology OR (prevalence or incidence) AND trends AND Africa) AND African AND (risk factors or contributing factors or predisposing factors)	80

### Study selection

The database search, which initially focused on the titles of the articles, was initially conducted by the first reviewer. The reviewer adhered to the inclusion and exclusion criteria during this process. Potentially relevant articles were obtained in full texts. These articles were exported to EndNote library version X8.2 [12] and duplicates removed. Two independent reviewers then conducted abstracts and full article screening. Disputes arising between the two reviewers were resolved by involving a third reviewer.

Studies were included if they met the following eligibility criteria:

### Eligibility criteria

- Quantitative primary studies focused on all types of pediatric cancers.
- Studies reporting evidence of pediatric cancer in the SSA region.
- Studies presenting evidence on the factors that are associated with the risk of pediatric cancers among children younger than 15 years, including studies with comparisons between exposed children and unexposed children (cohort and case-control studies).
- Articles presenting evidence on cancers among children aged below 15 years.

### Exclusion criteria

Studies reporting evidence on cancers among people aged 15 years and above were excluded. Studies presenting evidence conducted outside of SSA, as well as those presenting the approach of qualitative data were excluded.

### Charting data

Data was extracted using an electronic data extraction tool from Google forms. The following study characteristics and elements were extracted: author and year of publication, study title, study aim, study design, study population, sex, geographical setting, summary of findings/outcomes, country, and study limitations. Outcomes analyzed included type of risk factor and type of cancer.

### Summary and collating

After charting the data, a descriptive overview of the studies was presented in a narrative format. An account of the number of studies, research methods used, sex, types, and risk associated with pediatric cancers and outcomes was described in this narrative. The analysis was mainly based on mapping the country and disease-specific outcomes emerging from the studies.

## Results

**Fig. 1:**
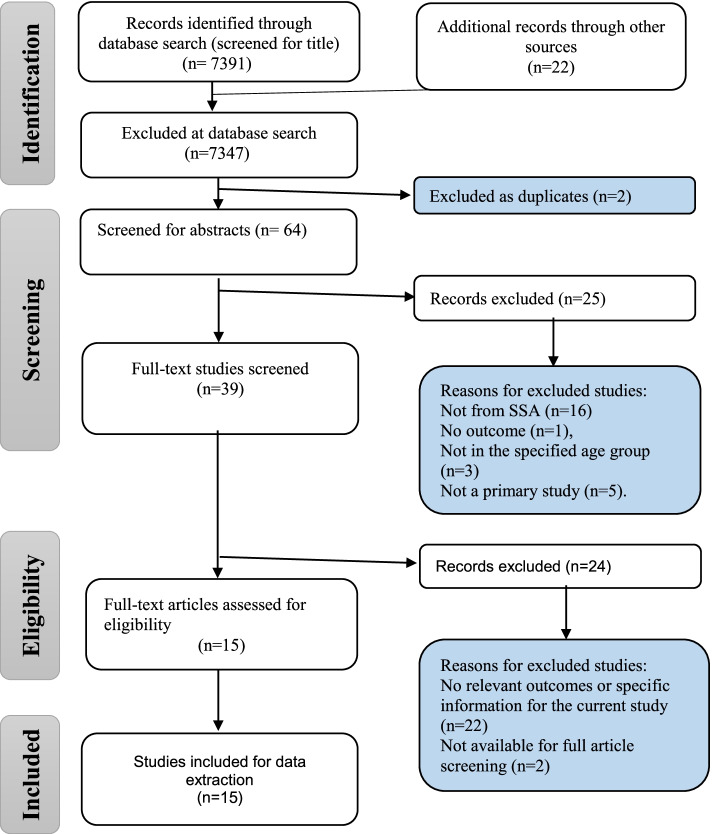
PRISMA flow diagram

The electronic search strategy identified 7391 references (Fig. 1), which were screened for titles. In addition, there were 22 articles retrieved by hand search and through database articles, references search. A total of 7347 articles were not selected during the database search stage because they did not meet the inclusion criteria. Two duplicates were removed, leaving 64 articles which were screened for abstracts [13–74]. A total of 25 articles were removed at the abstract screening stage because they formed part of the exclusion criteria (i.e., those conducted outside of SSA, those with no relevant outcomes for the study, those not in the specified age group, and those which are not primary studies). The researchers further screened 39 full-text articles and excluded 24 articles for the following reasons: 22 had no relevant outcomes for the current study and 2 were not available for full article screening. Therefore, 15 articles met our inclusion criteria.

### Overview of studies

This review included 15 studies that highlight risk factors for pediatric cancers [14, 27–29, 36, 53–59, 61, 73, 75]. Overall, there was limited evidence on the risk factors associated with pediatric cancers in SSA. A substantially small portion of SSA countries gave evidence on the risk factors associated with pediatric cancer (*n*=6). Evidence from the retrieved studies suggests that most childhood cancers in the SSA region are infection induced (*n*=12) [14, 27, 28, 36, 53, 55, 57–59, 61, 73, 75]. The type of cancer mostly reported is Burkitt Lymphoma and is diagnosed mostly the tropical region of SSA. Some studies did not provide a more comprehensive way of childhood cancer classification, making it difficult to discern which cancer was associated with a certain risk factor. The study designs employed were retrospective cohort (*n*= 9) and case-control studies (*n*= 6). The types of risk factors reported in the studies were not the same and for better ascertainment, we loosely divided the type of risk factors into three types: infection induced, genetic, and demographic risk factors. A more detailed summary of the included studies is described in Table 2.

**Table 2:** Characteristics of included studies

Author and date	Country and Period	Study aim	Study design	Study population characteristics			Outcome	Risk factor
				Population and age group	F%	M%		
*Moore et al. 2008* [27]	South Africa (1988-2006).	To assess recent changes in the prevalence and surgical management of liver tumours in South African children.	Retrospective chart review.	274 Children with liver tumours (0-14 years) from South African Children’s Cancer Study Group (SACCSG) Tumour Registry and as well as the individual POUs)	45.9	54.1	• Hepatoblastoma presented at a mean age of 2.16 years and was not identified in children over 4 years. • HCC was predominant in older patients (mean age: 10.48 years) but 6% was presented in children younger 8 years of age. • There was an increase in the incidence of vascular tumours, probably due to an increase in Kaposi-like sarcoma in retrovirus -positive patients.	Age Gender Ethnicity • 94 % of the children with HCC were black. • In 69% of HCC patients elevated Hepatitis B titres were identified. • Hepatitis B vaccination led to an observed decrease in the incidence of HCC. • There was a higher percentage of males indicating HBsAg predominance in males. Infections (Hepatitis B)
*Mulama et al. 2014* [28]	Kenya	To examine whether sickle cell trait, which is associated with protection from severe malaria and hyper-parasitaemia, decreases endemic BL risk. Secondary aim was to measure peripheral EBV load as an indicator of viral control and associate levels with HbAA/AS genotype	Case control study	306 children with a confirmed diagnosis of eBL and 537 geographically defined and ethnically matched controls.	40	60	• Sickle cell trait does not protect against eBL for children residing in malaria holoendemic areas after controlling for ethnicity. • Although not in line with the aim this study indirectly suggests that Epstein Barr Virus and Plasmodium falciparum coinfections are contributing risk factors to endemic Burkitt lymphoma (BL).	Infection (EBV and malaria)
*Rainey et al. 2007* [36]	Kenya (1988-1997)	To determine if strong associations between BL and malaria transmission remained when analysed using newer definitions of malaria transmission intensity and higher resolution maps than previously available.	Retrospective chart review and prospective study	665 Paediatric lymphoma cases (0-15years)			• Incidence rates varied by malaria transmission intensity in the different regions. • A positive trend between BL incidence rates and malaria transmission intensity was observed, supporting an etiologic role of malaria in BL oncogenesis. • In a log-linear model, BL rates were 3.5 times greater in regions with chronic and intense malaria transmission intensity than in regions with no or sporadic malaria transmission (OR = 3.47, 95% CI = 1.30–9.30), regardless of tribe.	Infection (malaria)
*Mwanda et al. 2004* [29]	Kenya (1988-1997)	To show the geographical (Provincial), age, gender and ethnic distribution of Burkitt's lymphoma in patients in Kenya.	Retrospective records review and prospective evaluation of patients	961Children with (0-14 years)	40	60	• The disease distribution is consistent with intermediate risk Burkitt's lymphoma level. • Furthermore the distribution varied by province, tribe, age and gender. • The variations could be due to environmental factors.	Environmental factors Demographic factors
*Makata et al. 1996* [56]	Kenya (1979-1994).	To review histologically the paediatric solid malignant tumours in western Kenya, with a focus on the following specific objectives: to examine tumours incidence, age, sex, geographic, and ethnic distribution and to relate the tumours to putative environmental or genetic causative factors	Retrospective study	600 Children with cancer (0-14years)	38	62	• Significantly high crude incidence rates for lymphomas and Kaposi's sarcoma showed a characteristic ethnogeographic distribution. • The majority of the tumors were found concentrated around Lake Victoria and showed decreasing occurrence as one moved towards the semi-arid and highland areas. • Lymphomas and Kaposi Sarcoma were most significant in humid and hot areas where malaria is hyperendemic. • Environmental factors seem to play a major role in childhood tumors in western Kenya. • BL, Non-Hodgkin Lymphoma (NHL ) and Kaposi Sarcoma (KS) had a high incidence in the Luo tribe as compared to other tribes in the same area. • Incidence was high in the 5-9-year age range (0.8/100,000/year) • Retinoblastoma and nephroblastoma occurred during early childhood (0-4years) and had a possibility of being hereditary. • BL incidence decreased with age whilst NHL and Hodgkin’s disease increased with age	Age Tribe Environmental factors Genetic factors
*Newton et al. 2001* [59]	Uganda (From march 1995 to February 1999)	To examine the association between HIV infection and various cancers using data from a case-control study of adults and children with cancer in Kampala, Uganda.	Case control study	128 Children with cancer and 190 children as controls (0-14 years).	40	60	• HIV infection was associated with a significantly increased risk of Kaposi’s sarcoma (OR = 94.9, 95% CI 28.5–315.3, based on 36 cases) and Burkitt’s lymphoma (OR = 7.5, 95% CI 2.8–20.1, based on 33 cases) but not with other cancers.	Infection (HIV) (EBV)
*Parkin et al. 2000* [62]	Uganda. (1994-1998).	To investigate the types of non-Hodgkin lymphoma (NHL) occurring in Kampala, Uganda, their association with Epstein-Barr virus (EBV), and how their risk is modified by HIV and other variables	Case control study	61 Children with non-Hodgkin 707 lymphoma (0-14 years)	29.2	70.8	• The vast majority of NHL cases in children were endemic BL (90%). • 51 of the 56 BL cases were tested for the presence of EBV and all were positive • EBV was not detected in the five cases of other lymphoma subtypes. • The risk in endemic BL was not modified by HIV with only 5.4 % of HIV positive cases in BL.	Infection (EBV)
*Ziegler and Mbidde, 1996* [63]	Uganda (1989-1994)	To report a retrospective analysis of 100 cases of KS in Ugandan children in the 6-year period	Retrospective review	100 Children with Kaposi sarcoma (0-15 years)	37	63	• The median age was 4 years. • The incidence of childhood KS increased more than 40-fold in the era of AIDS. • 78% of 63 cases tested were seropositive for HIV-1. • The increase of childhood KS implies that the prevalence of causative factors is rising in Uganda.	Infection (HIV)
*Aka et al. 2012* [54]	Tanzania (2000-2009)	To update baseline epidemiology of BL in northern Tanzania using recent data	Retrospective study	944 Children with BL (0-15 years)	42	58	• Tumors occurred at a younger mean age in boys than girls (6.8 years vs. 7.6 years, *P* < 0.01). • Crude BL incidence varied by region (3.0 in Mwanza vs. 6.8 in Mara), by district (1.4–22), by gender (5.0 in boys vs. 4.0 in girls), and by age group (2.0 in 0–4years, 7.8 in 5–9years, and 3.1 in 10–15 years).	Age Sex Ethnicity
*Amir et al. 2001* [14]	Tanzania (1968-1995)	To compare data on Kaposi Sarcoma in prepubescent children (0-14 years) recorded in the Tanzanian cancer registry before and during the AIDS epidemic	Retrospective chart review	150 Children with Kaposi sarcoma (0-14)	16	84	• Overall 73 (4.9/year) of these cases were registered during the pre and 77 (5.9/year) during the AIDS period. • The highest occurrence of PKS was observed in the 0- to 5-years age group. • The results imply that children younger than 5 years are at high risk of developing Kaposi Sarcoma. • Possibly reflecting low resistance to human herpes virus (HHV) 8 infection. • There is a likely hood that the increased susceptibility to HHV8 infection and morbidity is related to progressive immunodeficiency • The increase in AIDS PKS incidence appears to reflect a direct or indirect promoting effect of HIV on the development of KS lesions.	Age Infection (HIV and human herpes virus)
*Mutalima et al. 2008* [57]	Malawi	To report risk factors for childhood BL from a case-control study conducted in Malawi focusing particular attention on three infections: HIV, EBV and malaria.	Case control study	148 children with BL and 104 controls (children admitted with non-malignant conditions other than haematological malignancies and Kaposi Sarcoma (0-15yrs).	40	60	• Reported use of mosquito nets was associated with a lower risk of BL (OR = 0.2, 95% CI, 0.03 to 0.9, *p* = 0.04). • Among HIV negative participants, cases were thirteen times more likely than controls to have raised levels of EBV antibodies (OR=13.2; 95% CI 3.8 to 46.6; *p* = 0.001). • Cases were more likely than controls to be HIV positive OR = 12.4, 95% CI 1.3 to 116.2, *p* = 0.03). • ORs for BL increased with increasing antibody titers against EBV (*p* = 0.001)	Infection (EBV and malaria)
*Mutalima et al. 2010* [58]	Malawi (2005-2008)	To examine the association between HIV infection and cancer among children in Blantyre, Malawi	Case control study	541 Children with cancer (0-15years)	41	59	• 97% of the children were tested for HIV and 10% were found to be seropositive for HIV. • HIV infection was associated strongly and positively with Kaposi sarcoma (29 cases; OR = 93.5, 95% CI 26.9 to 324.4) and positively with non-Burkitt, non-Hodgkin lymphoma (33 cases; OR = 4.4, 95% CI 1.1 to 17.9).	Infection (HIV)
*Sinfield et al. 2007* [61]	Malawi 1998-2003).	To document the frequency of paediatric cancers presenting to a large central hospital in Malawi, detailing the presenting features, initial investigations, and HIV status of these children. (	Retrospective study	707 Children with cancer (0-15 years)			• There was a significant difference in the presentation of HIV- seropositive and seronegative children in the case of BL, non-Hodgkin lymphoma, and Kaposi sarcoma. • Kaposi sarcoma markedly increased in frequency over time. • The proportion of children with cancer who were tested for HIV increased over time but varied by cancer type. • Amongst those tested, the seroprevalence was 93% for children with Kaposi sarcoma, 4% (11/289) for those with BL, 31% for those with other non- Hodgkin lymphomas, 7% or those with Hodgkin disease, and 5% for those with other cancers.	Infection with HIV
*Chintu et al. 1995* [55]	Zambia (1980-1992)	To study the effect of the HIV epidemic on the epidemiology of cancers in children during the pre-HIV epidemic period and after the epidemic was established.	Retrospective study	698 Children with cancer (0-14 years)	36.8	63.2	• A significant increase in the occurrence of total childhood cancers was found. • This was mostly due to a highly significant increase in the incidence of paediatric Kaposi's sarcoma (*p* = 0.000016), which is causally related to HIV infection. • A prospective in-depth epidemiological study of HIV related childhood cancers in Africa is urgently needed.	Infection (HIV)

### Infection induced

There seemed to be an increased rate of childhood malignancies due to infections. Infection with HIV, hepatitis B, human herpes virus, EBV, and malaria were mainly associated with an increased risk of cancer in children. HIV (*n*=6) [14, 55, 58, 59, 61, 76] and EBV (*n*=5) [28, 36, 57, 59, 75] were the most reported infection induced risk factors in children with cancer.

Infection with HIV was associated with an increased risk of pediatric cancer, particularly in SSA. In a study from Zambia conducted two decades ago, HIV was casually related with the risk of getting Kaposi sarcoma in children [55]. This was also the case in studies from Malawi that were an increased incidence in Kaposi sarcoma was noted over time. The HIV seroprevalence was 93% for children with Kaposi sarcoma, 4% for those with Burkitt lymphoma, 31% for other non-Hodgkin lymphomas, 7% for Hodgkin disease, and 5% for other cancers [61]. In the second study, 10% were seropositive for HIV and HIV infection was strongly associated with Kaposi sarcoma and positively associated with non-Burkitt, non-Hodgkin lymphoma [58]. A similar trend was also observed in Uganda for pediatric Kaposi sarcoma [53, 59] and Burkitt lymphoma [59]. In Tanzania infection with human herpes virus (HHV8) and HIV reflected an indirect effect or direct effect on the development of Kaposi sarcoma lesions [14]. In a recent study from four countries Botswana, Malawi, Tanzania, and Uganda, it was observed that late ART initiation and severe immunosuppression led to a higher risk of cancer among children living with HIV [73]. Kaposi sarcoma in particular had a high incidence among children living with HIV [73].

Two case-control studies from Kenya and Malawi reported on the Joint pathogenesis of EBV and malaria in Burkitt Lymphoma [28, 57]. Burkitt lymphoma rates were observed to be 3.5 times (using log- linear model) higher in areas with intense malaria transmission as compared to regions with no malaria transmission [36]. Infection with malaria was positively linked to the oncogenesis of Burkitt lymphoma (odds ratio = 3.47, 95% confidence interval = 1.30–9.30) [36]. In another study from Uganda, 91% of endemic Burkitt lymphoma cases tested positive for EBV [75], reflecting a positive association between Burkitt lymphoma and EBV. Hepatitis B was associated with an increased risk of hepatocellular carcinoma in a retrospective study conducted in South Africa [27]. An observed decrease in the incidence of the latter was observed after vaccination for hepatitis B [27].

### Demographic

The mean age at diagnosis varied depending on the type of cancer and this was the trend seen in most of the articles reviewed [14, 27, 54, 56, 57]. The mean age at diagnosis for hepatoblastoma was 2.16 and was not diagnosed in children over 4 years. In the same study, hepatocellular carcinoma was common in older patients (mean age 10.48 years) [27].

In patients with Burkitt lymphoma, the mean age at diagnosis ranged from 5 to 7.6 years [54, 56, 57]. In a study from Kenya, it was noted that the Burkitt lymphoma incidence decreased with age [56] and in Tanzania the mean age at diagnosis varied between male and female patients (6.8 and 7.6 years, respectively) [54]. Retinoblastoma and nephroblastoma had a mean age diagnosis of 0–4 years [56], and pediatric Kaposi sarcoma was diagnosed at 0–5 years [14].

This review highlighted the varying levels of disease predominance or, in some cases, causative agents for a particular cancer among female or male patients [27]. For example, there was a higher predominance of male children with Burkitt lymphoma and Kaposi sarcoma in some of the included studies [29, 55]. Overall, there was a high percentage of males diagnosed with childhood cancer in comparison to females.

Pediatric cancer incidence and risk factors seemed to also vary by ethnicity or race. Of the children diagnosed with hepatic cellular carcinoma in South Africa, 94% were black [27]. Among the different tribes in the western Kenya region, the Luo tribe had the highest incidence of Burkitt lymphoma, non-Hodgkin lymphoma, and Kaposi sarcoma [56]. In northern Tanzania the crude incidence varied by region (3.0 in Mwanza and 6.8 in Mara) indicating variations in ethnicity [54]. In a study by Mwanda et al., they suggested that the observed demographic and geographic Burkitt lymphoma rate variations could be due to environmental factors [29]. Disease distribution was consistent with intermediate risk to Burkitt lymphoma level [29], and in another study by Makata et al., a large number of tumors were found concentrated around Lake Victoria and decreased in occurrence as one moved to semi-arid and highland areas [56]. Lymphomas were significant in humid and hot areas where malaria is hyperendemic [56].

## Discussion

Children in SSA diagnosed with cancer have documented risk factors marked by sex, ethnic, and age disparities, and these variations have been long observed [3]. We classified the latter as demographic factors. Although there was no consistent evidence to prove that they contribute to the causality of cancer in children, the trend was that certain cancers where predominant or occurred at a certain age, ethnicity, and sex. The age at onset varies for various cancers, and the latency periods of different cancers also vary [6]. Most of the malignancies seemed to peak at a young age, but the reason was unknown or obscure. Malignancies like nephroblastoma, neuroblastoma, and brain tumors’ peak in infancy and acute lymphoblastic leukemia peaks at 2–4 years [3, 6]. It is thought that they may be related to prenatal exposures and those that occur in adolescents like renal carcinoma may be linked to hormonal changes that occur in the body [6]. There was no data on the risk associated with parental age at conception; however, in studies from HICs, there has been disparities in the role of parental age at conception. Whilst some authors reported an increase in the risk of pediatric cancer with increased maternal age [77–79], others concluded that younger maternal age increased the risk of pediatric cancer [7, 80, 81].

There was an overall male predominance for a majority of the pediatric cancers in the reviewed articles [14, 29, 54–59, 75, 76]. This trend was also observed from the incidence rates/million for international classification of childhood cancer categories by sex [82]. The incidence differences that exist between males and females are linked to various exposures that differ by sex. Moreover, it is important to note that hormonal effects and genetic disparities between boys and girls exist [83]. High incidence among males is observed in cancers like non-Hodgkin’s lymphoma, Hodgkin’s disease, and acute lymphoblastic lymphoma [83]. A higher incidence exist among females for thyroid carcinoma and malignant melanoma [83].

In SSA, it was clear to see that some cancers vary by ethnicity or race, this is marked by the differing cancer rates in different geographic areas like Kenya and Tanzania. In high-income countries, the incidence of most types of cancer is lower in Black, Hispanic, and Asian children as compared to white children and the disparities are quiet dramatic [82]. With the exception of one article [27], there was not much data from the reviewed articles for us to determine the racial trends in the SSA region. Hepatocellular carcinoma was found to be more predominant in the black childhood population in South Africa. There is ethnic variability in the pathogenesis of hepatocellular carcinoma as hepatitis C virus was implicated as the cause of HCC among White and Black individuals whereas hepatitis B was the cause among the Asians in the US community [84]. In Africa, there is a wide variation among the population with respect to genes and environments [3]. It therefore follows that human cancer patterns vary and are diverse. The degree to which racial or ethnic factors are attributable to genetic versus environmental differences has not been determined [3].

Environmental exposures play a major role in the cause of childhood cancers. In this review, infectious agents or biological carcinogens were found to increase the risk or cause cancer in children. Infections are the causes of commonly diagnosed cancers in Africa, for example cervix, liver and bladder cancers and Kaposi sarcoma [85]. Infection with hepatitis B is a known cause of hepatocellular carcinoma [86]. HIV has also been long known to increase the risk of cancer like Kaposi sarcoma and non-Hodgkin lymphoma, and these cancers are known as AIDS defining cancers [87, 88]. An increased risk in Kaposi sarcoma has been observed in HIV-positive individuals infected with human herpes virus-8 and is acquired horizontally from mothers [87].

Infection with Epstein-Barr virus (EBV) in children in Africa occurs soon after birth and intense infection with malaria is an important cofactor in the pathogenesis of Burkitt lymphoma [87]. Malarial holo-endemicity has been associated with the geographical distribution of Burkitt lymphoma [89, 90]. EBV is said to be detectable in 95% of childhood endemic Burkitt lymphoma cases [87]. A cure or vaccination proves the cause to a disease. In the current review, some articles mentioned causes to the abovementioned malignancies. An example is the decrease in the incidence of HCC after vaccinating for hepatitis B in South African children [27] and a decrease in the incidence of Burkitt lymphoma in some parts of Africa was attributed to the introduction of antimalarial programs [90].

### Strengths and limitations of the study

Overall, we were able to summarize what is known and gaps on the risk factors associated with pediatric cancers. Thorough and comprehensive literature search was carried out, and to minimize bias, there was no language and date restriction. Complete and transparent presentation of the results was ensured through the employment of the PRISMA flow diagram.

In this review, most articles used two main study designs: retrospective and case-control studies. According to Linet et al., an ideal case-control study is one that is nested within a cohort (prospective or retrospective), in which all cases are ascertained, but a randomly selected sample of the cohort is used for controls [6]. Since pediatric cancers are rare, it is justified economically that retrospective studies were employed although most cases are a subject to selection bias.

Caution should however be taken when analyzing or interpreting this data as some studies did not provide a more comprehensive way of childhood cancer classification, making it difficult to discern which cancer was associated with a certain risk factor. It was also difficult to group some risk factors into a certain typology as there was an overlap between environmental factors and demographic factors for example in studies by Makata et al. [56] and Mwanda et al. [29].

Although the methodology was followed with accuracy, the search results might be subject to bias as some studies might have been excluded due to the inclusion or exclusion of certain search terms. There was a deviation from the published protocol as the study did not report on the main research question that sought to find the types of evidence and concepts behind estimating the distribution of pediatric cancers in SSA. This study did not also address the trends in pediatric cancer in relation to HIV in SSA.

### Implications for research and practice

Current evidence on what has been done will create baseline knowledge on what to focus on; however, knowledge on its own is not sufficient to bring change in cancer management in the SSA region. Some cancers were diagnosed at a young age, and further research needs to be conducted on susceptible immunity and the role of prenatal factors at a young age leading to exposure. Furthermore, there is need for more current studies, coupled with the collection of biological samples as compared to records review. This will ensure the measurement of known and unknown cancer risk factors, monitoring their gradual development and assessing the effectiveness of prevention and treatment interventions.

The use of standardized methods in the measuring of exposures should be well thought of. This is important in the reproducibility of measurements over time, data validity, and accuracy of exposure measurements. The other advantage of using standardized methods for measuring exposure is that adequate quality control, and data collection protocols can be incorporated.

## Conclusion

The review aimed to map evidence on the risk factors associated with pediatric cancers in SSA. Based on the weight of evidence obtained and taking into consideration the general rarity of these cancers with the exception of exposure to infections, there seemed to be limited evidence. The limited evidence on the risk factors of these malignancies in the SSA hampers efforts to set priorities for childhood cancer control. Formulation of effective preventative (where possible) measures and treatment regimens will need proper assessment of risk factors.

## Data Availability

The data reported and supporting this paper was sourced from the existing literature, therefore are available through the detailed reference list.

## Abbreviations

EBV: Epstein-Barr virus
HCC: Hepatic cellular carcinoma
LMICs: Low- to medium-income countries
LIC: Low-income countries
MeSH: Medical Subject Headings
MMAT: Mixed methods appraisal tool
HIV: Human immunodeficiency virus
HIC: High-income countries
AIDS: Acquired immunodeficiency syndrome
PRISMA: Preferred Reporting Items for Systematic Reviews and Meta-analysis
SSA: Sub-Saharan Africa
WHO: World Health Organization

## References

[1] Buka I, Koranteng S, Osornio Vargas AR (2007). Trends in childhood cancer incidence: review of environmental linkages. Pediatr Clin N Am.

[2] Magrath I (2013). Paediatric cancer in low-income and middle-income countries. Lancet Oncol.

[3] Spector LG, Pankratz N, Marcotte EL (2015). Genetic and nongenetic risk factors for childhood cancer. Pediatr Clin N Am.

[4] Scheurer ME (2018). An overview of disparities in childhood cancer: report on the Inaugural Symposium on Childhood Cancer Health Disparities, Houston, Texas, 2016. Pediatr Hematol Oncol.

[5] Degar B, Isakoff M, Zaoutis LB, Chiang VW (2007). Chapter 123 - childhood cancer. Comprehensive pediatric hospital medicine.

[6] Linet MS, Wacholder S, Zahm SH (2003). Interpreting epidemiologic research: lessons from studies of childhood cancer. Pediatrics.

[7] Bhattacharya S (2014). Maternal and perinatal risk factors for childhood cancer: record linkage study. BMJ Open.

[8] Alibek K (2013). Childhood cancers: what is a possible role of infectious agents?. Infect Agents Cancer.

[9] Renner L (2018). Evidence from Ghana indicates that childhood cancer treatment in sub-Saharan Africa is very cost effective: a report from the childhood cancer 2030 network. J Glob Oncol.

[10] Galárraga O (2009). HIV prevention cost-effectiveness: a systematic review. BMC Public Health.

[11] Ndlovu SR, Kuupiel D, Ginindza TG (2019). Mapping evidence on the distribution of paediatric cancers in sub-Saharan Africa: a scoping review protocol. Syst Rev.

[12] Endnote. Available from: https://softwarerep.ukzn.ac.za/Academic%20Software/EndNote/. Accessed 15 Sept 2020.

[13] Akang E (1996). Tumors of childhood in Ibadan, Nigeria (1973-1990). Pediatr Pathol Lab Med.

[14] Amir H (2001). Kaposi’s sarcoma before and during a human immunodeficiency virus epidemic in Tanzanian children. Pediatr Infect Dis J.

[15] Athale UH (1995). Influence of HIV epidemic on the incidence of Kaposi's sarcoma in Zambian children. J Acquir Immune Defic Syndr Hum Retrovirol.

[16] Dauda MA (2014). Sarcomas in Nigerian children in Jos North Central Nigeria. Afr J Med Med Sci.

[17] Davidson A (2014). Malignancies in South African children with HIV. J Pediatr Hematol Oncol.

[18] Ekenze SO (2009). The burden of pediatric malignant solid tumors in a developing country. J Trop Pediatr.

[19] Fraumeni JF, Miller RW, Hill JA (1968). Primary carcinoma of the liver in childhood: an epidemiologic study. J Natl Cancer Inst.

[20] Greaves MF (1997). Aetiology of acute leukaemia. Lancet.

[21] Gupta S (2014). Pediatric oncology as the next global child health priority: the need for national childhood cancer strategies in low-and middle-income countries. PLoS Med.

[22] Hadley LG (2012). Challenge of pediatric oncology in Africa. Semin Pediatr Surg.

[23] Kerr DA, Kaschula ROC (2013). Pediatric pathology services in Africa. Arch Pathol Lab Med.

[24] Kramárová E, Stiller CA (1996). The international classification of childhood cancer. Int J Cancer.

[25] Kruger M (2014). Childhood cancer in Africa. Pediatr Blood Cancer.

[26] McBride ML (1998). Childhood cancer and environmental contaminants. Can J Public Health.

[27] Moore SW (2008). Malignant liver tumors in South African children: a national audit. World J Surg.

[28] Mulama DH (2014). Sickle cell trait is not associated with endemic Burkitt lymphoma: an ethnicity and malaria endemicity-matched case–control study suggests factors controlling EBV may serve as a predictive biomarker for this pediatric cancer. Int J Cancer.

[29] Mwanda OW, et al. Burkitt’s lymphoma in Kenya: geographical, age, gender and ethnic distribution. East Afr Med J. 2004;(8 Suppl):S68–77. 10.4314/eamj.v81i8.9210. 10.4314/eamj.v81i8.9210 15622605

[30] Ochicha O, Gwarzo AK, Gwarzo D (2012). Pediatric malignancies in Kano, northern Nigeria. World J Pediatr.

[31] Ojesina AI, Akang EEU, Ojemakinde KO (2002). Decline in the frequency of Burkitt’s lymphoma relative to other childhood malignancies in Ibadan, Nigeria. Ann Trop Paediatr.

[32] Orem J, Otieno MW, Remick SC (2004). AIDS-associated cancer in developing nations. Curr Opin Oncol.

[33] Parkin DM (2008). Part I: Cancer in Indigenous Africans--burden, distribution, and trends. Lancet Oncol.

[34] Pollock BH (2003). Risk factors for pediatric human immunodeficiency virus–related malignancy. JAMA.

[35] Pritchard-Jones K (2013). Sustaining innovation and improvement in the treatment of childhood cancer: lessons from high-income countries. Lancet Oncol.

[36] Rainey JJ (2007). Spatial distribution of Burkitt’s lymphoma in Kenya and association with malaria risk. Tropical Med Int Health.

[37] Smith MA (1998). Evidence that childhood acute lymphoblastic leukemia is associated with an infectious agent linked to hygiene conditions. Cancer Causes Control.

[38] Stefan D (2015). Childhood cancer incidence in South Africa, 1987–2007. S Afr Med J.

[39] Stefan DC (2015). Patterns of distribution of childhood cancer in Africa. J Trop Pediatr.

[40] Stefan DC (2015). Childhood cancer in Africa: an overview of resources. J Pediatr Hematol Oncol.

[41] Stefan DC, Baadjes B, Kruger M (2014). Incidence of childhood cancer in Namibia: the need for registries in Africa. Pan Afr Med J.

[42] Stefan DC, Lutchman R (2014). Burkitt lymphoma: epidemiological features and survival in a South African centre. Infect Agents Cancer.

[43] Stinton LM, Shaffer EA (2012). Epidemiology of gallbladder disease: cholelithiasis and cancer. Gut Liver.

[44] Stones DK (2014). Childhood cancer survival rates in two South African units. S Afr Med J.

[45] Usman B, Mohammed A (2015). Carcinoma in children at Ahmadu Bello University Teaching Hospital Zaria. Ann Niger Med.

[46] Ward E (2014). Childhood and adolescent cancer statistics, 2014. CA Cancer J Clin.

[47] Weaver MS (2015). The prioritisation of paediatrics and palliative care in cancer control plans in Africa. Br J Cancer.

[48] Wessels G, Hesseling PB (1997). Incidence and frequency rates of childhood cancer in Namibia. S Afr Med J.

[49] Wiredu EK, Armah HB (2006). Cancer mortality patterns in Ghana: a 10-year review of autopsies and hospital mortality. BMC Public Health.

[50] Wrensch M (2002). Epidemiology of primary brain tumors: current concepts and review of the literature. Neuro-Oncol.

[51] Kruger M (2014). Retinoblastoma outcome at a single institution in South Africa. S Afr Med J.

[52] Athale UH (1995). HIV epidemic on the Influence of incidence of Kaposi’s sarcoma in Zambian children. J Acquir Immune Defic Syndr Hum Retrovirol.

[53] Ziegler JL (1997). Risk factors for Kaposi’s sarcoma in HIV-positive subjects in Uganda. Aids.

[54] Aka P (2012). Incidence and trends in Burkitt lymphoma in northern Tanzania from 2000 to 2009. Pediatr Blood Cancer.

[55] Chintu C, Athale UH, Patil P (1995). Childhood cancers in Zambia before and after the HIV epidemic. Arch Dis Child.

[56] Makata AM (1996). The pattern of pediatric solid malignant tumors in western Kenya, east Africa, 1979–1994: an analysis based on histopathologic study. Am J Trop Med Hyg.

[57] Mutalima N (2008). Associations between Burkitt lymphoma among children in Malawi and infection with HIV, EBV and Malaria: results from a case-control study. PLoS One.

[58] Mutalima N (2010). Impact of infection with human immunodeficiency virus-1 (HIV) on the risk of cancer among children in Malawi - preliminary findings. Infect Agents Cancer.

[59] Newton R (2001). A case-control study of human immunodeficiency virus infection and cancer in adults and children residing in Kampala, Uganda. Int J Cancer.

[60] Parkin DM, Stefan C (2017). Editorial: childhood cancer in sub-Saharan Africa. ecancermedicalscience.

[61] Sinfield RL (2007). Spectrum and presentation of pediatric malignancies in the HIV era: experience from Blantyre, Malawi, 1998–2003. Pediatr Blood Cancer.

[62] Ferndale L (2020). Gender differences in oesophageal squamous cell carcinoma in a South African Tertiary Hospital. Int J Environ Res Public Health.

[63] Harlemon M (2020). A custom genotyping array reveals population-level heterogeneity for the genetic risks of prostate cancer and other cancers in Africa. Cancer Res.

[64] Mohosho MM (2021). HIV prevalence in patients with cervical carcinoma: a cohort study at a secondary hospital in South Africa. Medicine.

[65] Munishi OM (2020). Awareness of cancer risk factors and its signs and symptoms in Northern Tanzania: a cross-sectional survey in the general population and in people living with HIV. J Cancer Educ.

[66] Okeke E (2020). Epidemiology of liver cancer in Africa: current and future trends. Semin Liver Dis.

[67] Parkin DM (2020). Cancer in Africa 2018: the role of infections. Int J Cancer.

[68] Sadykova LR (2020). Epidemiology and risk factors of osteosarcoma. Cancer Investig.

[69] Vo Quang E, Shimakawa Y, Nahon P (2021). Epidemiological projections of viral-induced hepatocellular carcinoma in the perspective of WHO global hepatitis elimination. Liver Int.

[70] Peprah S (2020). Risk factors for Burkitt lymphoma in East African children and minors: a case-control study in malaria-endemic regions in Uganda, Tanzania and Kenya. Int J Cancer.

[71] Redmond LS (2020). Endemic Burkitt lymphoma: a complication of asymptomatic malaria in sub-Saharan Africa based on published literature and primary data from Uganda, Tanzania, and Kenya. Malar J.

[72] Kirimunda S (2020). Variation in the Human Leukocyte Antigen system and risk for endemic Burkitt lymphoma in northern Uganda. Br J Haematol.

[73] Haq H (2021). Association between antiretroviral therapy and cancers among children living with HIV in sub-Saharan Africa. Cancers.

[74] Apple A, Lovvorn HN (2020). Wilms tumor in sub-Saharan Africa: molecular and social determinants of a global pediatric health disparity. Front Oncol.

[75] Parkin DMA (2000). Non-Hodgkin lymphoma in Uganda: a case-control study. AIDS.

[76] Ziegler JL, Katongole-Mbidde E (1996). Kaposi’s sarcoma in childhood: an analysis of 100 cases from Uganda and relationship to HIV infection. Int J Cancer.

[77] Johnson KJ (2009). Parental age and risk of childhood cancer: a pooled analysis. Epidemiology (Cambridge, Mass).

[78] Maule MM (2007). Effects of maternal age and cohort of birth on incidence time trends of childhood acute lymphoblastic leukemia. Cancer Epidemiol Prev Biomarkers.

[79] Maule MM (2009). How the effect of maternal age on the risk of childhood leukemia changed over time in Sweden, 1960–2004. Environ Health Perspect.

[80] Johnson KJ (2008). Perinatal characteristics and risk of neuroblastoma. Int J Cancer.

[81] Schüz J (1999). Association of childhood cancer with factors related to pregnancy and birth. Int J Epidemiol.

[82] Steliarova-Foucher E (2005). International classification of childhood cancer. Cancer.

[83] Ries LAG, et al. Cancer incidence and survival among children and adolescents: United States SEER Program 1975-1995. 1999.

[84] Di Bisceglie AM (2003). Hepatitis C–related hepatocellular carcinoma in the United States: influence of ethnic status. Am J Gastroenterol.

[85] Jemal A (2012). Cancer burden in Africa and opportunities for prevention. Cancer.

[86] Bisceglie A, Rustgi V, Hoofnagle J, Dusheiko G, Lotze M (1988). Hepatocellular carcinoma. Ann Intern Med..

[87] Sitas F (2008). Part II: cancer in indigenous Africans--causes and control. Lancet Oncol.

[88] Sitas F (2000). The spectrum of HIV-1 related cancers in South Africa. Int J Cancer.

[89] Rainey JJ (2007). Spatial clustering of endemic Burkitt’s lymphoma in high-risk regions of Kenya. Int J Cancer.

[90] Geser A, Brubaker G, Draper CC (1989). Effect of a malaria suppression program on the incidence of African Burkitt’s lymphoma. Am J Epidemiol.

